# How do novices learn physical examination skills? A systematic review of the literature

**DOI:** 10.1080/10872981.2019.1608142

**Published:** 2019-04-29

**Authors:** Aaron R. Danielson, Sandhya Venugopal, Jason M. Mefford, Samuel O. Clarke

**Affiliations:** a Department of Emergency Medicine, University of California at Davis, Sacramento, CA, USA; b Division of Cardiovascular Medicine, University of California at Davis, Sacramento, CA, USA; c Department of Emergency Medicine, Kaiser Permanente, Santa Clara, CA, USA

**Keywords:** Systematic review, physical examination, medical student, preclinical, teaching

## Abstract

**Background**: Physical Examination (PE) skills are vital for patient care, and many medical students receive their first introduction to them in their pre-clinical years. A substantial amount of curriculum time is devoted to teaching these skills in most schools. Little is known about the best way to introduce PE skills to novice learners.

**Objective**: Our objective was to conduct a systematic review of how medical students are first taught PE skills and the evidence supporting these strategies.

**Design**: We searched ERIC, SCOPUS, MEDLINE, PubMed and EMBASE for descriptions of complete PE curricula for novice learners. Inclusion criteria were: (1) English language; (2) subjects were enrolled in medical school and were in the preclinical portion of their training; (3) description of a method to teach physical examination skills for the first time; (4) description of the study population; (5) Description of a complete PE curriculum. We used the Medical Education Research Study Quality Instrument (MERSQI) score to evaluate the quality of evidence provided.

**Results**: Our search returned 5,418 articles; 32 articles met our inclusion criteria. Two main types of curricula were reported: comprehensive ‘head-to-toe’ PE curricula (18%) and organ system-based curricula (41%). No studies compared these directly, and only two evaluated trainees’ clinical performance. The rest of the articles described interventions used across curricula (41%). Median MERSQI score was 10.1 Interquartile range 8.1–12.4. We found evidence for the use of non-faculty teaching associates, technology-enhanced PE education, and the addition of clinical exposure to formal PE teaching.

**Conclusions**: The current literature on teaching PE is focused on describing innovations to head-to-toe and organ system-based curricula rather than their relative effectiveness, and is further limited by its reliance on short-term outcomes. The optimal strategy for novice PE instruction remains unknown.

## Background

Physical examination (PE) skills are essential to the practice of clinical medicine. They are considered a core patient care competency as defined by the Accreditation Council for Graduate Medical Education, as well as the newer entrustable professional activities (EPAs) for medical students described by Chen et al. [1,2]. Generally, it is thought that developing strong PE skills among physicians both improve the quality and decreases the cost of healthcare [3]. Despite this belief in the value of PE, multiple studies have found that both undergraduate- and graduate-level trainees’ PE skills are lacking [4–9].

As part of a general needs assessment on PE skills, we sought the supporting evidence for common PE curricula used to teach medical students PE skills for the first time. Our search yielded a limited number of review articles. Mookherjee et al. describe the state of PE instruction in graduate medical education (GME) [10] and Easton et al. describe underlying educational theory in teaching PE skills in a narrative review [11]. Recently, Moßhammer et al. published a scoping review describing how PE is taught in general practice [12]. However, a systematic review of the literature describing approaches to teaching PE skills to novice medical students is currently lacking.

In our experience, there are two common ways novices are taught PE skills. First, a head-to-toe checklist. In this case, students perform all taught maneuvers starting at the patients’ head and move more caudally until all maneuvers have been performed. Alternatively, an organ-system-based approach, where exam maneuvers are grouped based on the organ system they examine. While Moßhammer et al. describe teaching methodology in their review they did not describe the overall curriculum design in which these methodologies were used. Their scope also included interventions applied to learners at all phases of undergraduate medical education training [12].

We therefore set out to conduct a systematic review of the literature regarding PE curricula used to teach these skills to novice learners. Since medical schools do not require clinical skills training as a prerequisite, we considered matriculating medical students to be novices at PE for the purpose of our review. In the traditional USA Curriculum, a student in the first two years of training would be considered ‘pre-clinical’ and there for a novice. However, increasingly this line is blurred with some schools having shortened curricula or rapidly integrating students into the clinical environment. Medical schools across the world may teach physical examination for the first time at a variety of time points after learners enter medical school. Our goal was to be as inclusive as possible of methods used to teach learners PE skills for the first time. We therefore defined any first-time learner of PE skills ‘pre-clinical’ since it would not be possible to conduct a clinical evaluation of a patient without these skills. We set out to answer the following questions:

1. How are PE skills taught to preclinical medical students for the first time and what evidence supports these curricula?
2. What is the quality of evidence on teaching preclinical medical students PE skills?
3. How do the head-to-toe and organ-system-based curricula perform in comparison to each other?
4. What interventions, such as readings or online modules, have been applied to introductory PE curricula, and what evidence supports their use?

## Methods

### Target population

The goal of our review was to find strategies used to teach preclinical medical students PE skills and to evaluate the quality of evidence supporting these methods. We defined pre-clinical students as those receiving PE training prior to participating in a clerkship or other clinical activity.

### Literature search

We searched ERIC, SCOPUS, MEDLINE, EMBASE and PubMed using a predefined search strategy (see Appendix A and Table 1). The search was conducted on 1/30/15. We included all studies published prior to the date of the search.

**Table 1.** Characteristics of 32 studies included in a systematic review of physical examination curricula used to teach novice learners published through january 2015.

Characteristic	No. (% of 32) Studies	Studies
**Publication year**		
Pre-1980	7/32	[22]
1980–1989	3/32	[9]
1990–1999	9/32	[28]
2000–2009	9/32	[28]
2010–2015	4/32	[13]
**Country**		
USA	Other	[75]
Other	8/32	[25]
**Organizational approach**		
Head-to-toe	6/32	[18]
Organ system	13/32	[41]
Not defined	13/32	[41]
**Study design**		
Single group	6/32	[18]
Two-group, nonrandomized, concurrent	4/32	[13]
Two-group, nonrandomized, non-concurrent	10/32	[31]
Two-group, randomized controlled trial	5/32	[16]
> 2-group, randomized controlled trial	3/32	[9]
Not described	4/32	[13]
**Learner Level**		
MS-1	14/32	[44]
MS-2	11/32	[33]
Combined MS-1 and −2	3/32	[9]
MS-3^a^	4/32	[13]

^a^MS = medical student. MS-3 studies involved preclinical students at schools outside of the USA.

### Eligibility criteria

We used the definition of ‘physical examination’ established by Walker et al. and used by Mookerjee et al.: ‘the process of evaluating objective anatomic findings through the use of observation, palpation, percussion, and auscultation’ [10,13]. Our goal was to maintain broad inclusion criteria to be able to find any methods used for teaching novice medical students PE skills. We evaluated each study for inclusion based on the following criteria: (1) English language; (2) subjects were enrolled in medical school and were in the preclinical portion of their training; (3) description of a method to teach physical examination skills for the first time; (4) description of the study population; (5) description of a curriculum that teaches a complete PE (as opposed to a single organ system or other small group of maneuvers). Since our goal was to describe the overall strength of evidence in the current literature, we did not set a quality cut-off as part of the inclusion criteria.

### Title and abstract review

All duplicates were removed from the initial literature search. Next, one investigator screened each abstract for criteria 1–3 above. All studies meeting these criteria were screened by two investigators for inclusion using criteria 1–5. If criteria for inclusion could not be ascertained by title and abstract review alone, the text of the publication was reviewed by two of the investigators. A third investigator adjudicated any discrepancies between the two investigators regarding the inclusion of an article.

### Study review

Included publications were reviewed by a minimum of two investigators. Study quality was evaluated using the Medical Education Research Study Quality Instrument (MERSQI), a previously validated quality instrument used in several systematic reviews of the medical education literature [10,14–16].

### Statistical analysis

Descriptive statistics for the MERSQI score were calculated for all articles. Statistical analysis was performed using SPSS 24 (IBM, Armonk, New York). We anticipated a high degree of heterogeneity between studies and therefore did not conduct a meta-analysis of the included studies.

## Results

### General description

**Figure 1.**
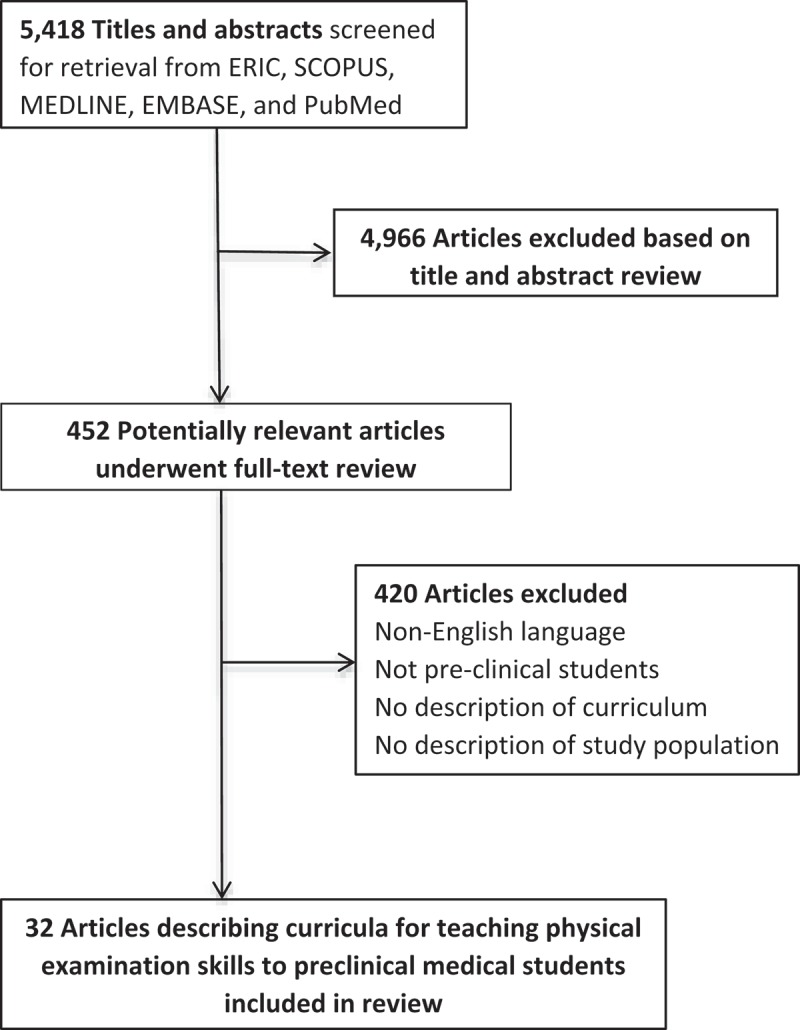
Selection process used in a systematic review of preclinical medical student physical examination curricula published through 1 January 2015.

Our initial search returned 5,418 articles. After our screening process we found 32 articles that met inclusion criteria for final review (Figure 1) [17–48].Table 1 summarize the characteristics of included studies (see Appendix B for detailed information of the studies and curricula included in the review). Publication dates ranged from 1978 to 2015. Studies from 10 countries were included. Studies included head-to-toe curricula (18%), organ system-based curricula (41%) or did not specifically define the type of curriculum used in these terms (41%). Undefined curricula discussed interventions added to an existing clinical skills curriculum. Since the intervention was described and applied to a complete curriculum, these studies were included. No studies specifically compared the head-to-toe approach to the organ system-based approach to teaching PE skills. No studies described the theoretical framework behind the decision to use a head-to-toe versus organ system-based curriculum or explained how the approach chosen translated to patient care.

The quality of the included studies varied widely. MERSQI scores for the included studies were not normally distributed (Shapiro-Wilk *P =*0.02). Scores ranged from 0.5 to 15. The median score was 11, with an interquartile range of 8.1–12.4. 25% of the included studies was randomized controlled trials [22,23,28,36,38,40,41,46].

### What questions were addressed?

The included studies addressed a number of questions, including:

1. When should PE skills be taught in pre-clinical curricula?
2. Can different types of instructors teach PE skills?
3. How effective are specific technologies at augmenting PE skill learning?
4. How useful is a specific organizational change in improving PE learning?

### Timing of instruction

Different institutions studied the teaching of PE skills at different times in the pre-clinical curriculum. 44% taught these skills to first-year medical students, and 33% taught them to second-year students. 13% of included studies taught the skills in the third year of medical school. The curricula introducing clinical skills in the third year were from Australia, Brazil, Japan and China, but all participants were pre-clinical students [30,34,38,47]. Two studies specifically addressed when in the pre-clinical curriculum to teach clinical skills [24,44]. Rogers et al. compared the teaching of clinical skills to second-year medical students to a curriculum that taught the same skills to first-year medical students [44]. They found that first-year students showed statistically superior performance on an Objective Structured Clinical Examination (OSCE). A second study by Davidson et al. indirectly addressed the same issue [24]. They performed a trial of a new curriculum taught to first-year students to an old curriculum taught to second-year students and compared the performance of both groups on an OSCE. The groups were compared on six stations (blood pressure measurement, eye, chest, abdominal, thyroid and cranial nerve examinations). They also found the superior performance of the first-year students on all but the eye and abdominal examinations. The second-year students involved had not received any clinical skills training. The curricula differed in terms of content. The first-year curriculum only taught normal PE findings, and the second-year curriculum taught normal and abnormal PE findings. No studies compared learner performance after pre-clinical PE training to PE training during the clinical years of medical school.

### Outcomes of curriculum effectiveness

Table 2 describes the outcomes used to determine the success of the included curricula. Outcomes included learner attitudes, OSCE scores, standardized patient (SP) encounters, written tests, instructor attitudes, and cost analysis. Two studies used clinical performance as an outcome of a pre-clinical curriculum [32,37]. Most studies with an objective performance measure used internally developed examinations as metrics. Cost analysis was a novel outcome used in three studies to describe the potential advantage of using non-faculty instructors to teach PE courses [17,24,31].

**Table 2.** Interventions and measured outcomes of 32 studies included in a systematic review of physical examination curricula used to teach novice learners published through January 2015.

Characteristic	No. (% of 32)	Studies
**Intervention**		
Use of non-faculty teaching associates^a^	11/32	[34]
Use of technology to teach PE skills^b^	5/32	[16]
Use of clinical setting to teach PE skills^c^	4/32	[13]
Change in organizational structure of curriculum	10/32	[31]
Description of existing curriculum (no intervention)	5/32	[16]
**Measured outcome**		
Learner attitudes	15/32	[47]
Instructor attitudes	3/32	[9]
Objective structured clinical exam (OSCE)	17/32	[52]
Standardized patient encounter	4/32	[13]
Clinical evaluation	2/32	[6]
Written tests^d^	4/32	[13]
Cost analysis	3/32	[9]

^a^Non-physician teaching associates included senior medical students, standardized patients, resident physicians and nurse practitioners. ^b^Technology included ultrasound, video and computer modules. ^c^Clinical setting included ambulatory, inpatient and nursing home settings. ^d^Written tests included multiple choice tests, clerkship exams and NBME shelf exams.

### Who can teach PE skills?

Eleven studies addressed the question of who can facilitate the teaching of PE skills [17,21–24,26,28,31,33,46,48]. The use of Physical Examination Teaching Associates (PETAs) was addressed in five studies [17,21,22,24,26]. PETAs were defined as laypersons trained in PE skills who then taught these skills to novice learners. In all cases, the students taught by PETAs showed either equivalent or superior performance to those taught by physicians. Four studies evaluated the effectiveness of more senior medical students to teach PE skills [23,28,33,48]. Two studies evaluated the effectiveness of second-year medical students as instructors in physical examination skills [33,48]. Kim et al. studied the use of second-year medical students to teach PE practice sessions in a novice curriculum and found that this improved learner comfort in performing PE skills [33]. Wasson et al. compared the performance of first-year medical students taught PE skills by second-year medical students to a control group of third-year medical students taught PE skills by faculty [48]. When compared in their performance of a complete history and physical, the first-year students were found to be statistically superior. The outcome used was thoroughness which was evaluated using a novel metric [48]. No validation of this metric was described. Barnes et al. compared groups of first-year students taught by fourth-year students to groups taught by faculty, fellows or residents and found no difference in student performance on a station in which learners were asked to perform the PE for a respiratory complaint as part of an OSCE [23]. Stillman et al. compared the ability of faculty, residents and nurse practitioners to teach a head-to-toe physical examination to different groups of medical students [46]. In this study, there was no statistical difference between groups as compared on the performance of a 210 maneuver head-to-toe PE.

### What technologies augment PE learning?

Five studies evaluated the addition of technology to PE courses for novices and studied the effect of these adjuncts on learning [25,36,38,41,42]. Mir et al. evaluated the replacement of live demonstration of physical examination with video demonstration and found that this resulted in no difference in student performance on a 5-station OSCE evaluating the performance of knee, abdomen, ‘motor function,’ thyroid and pulse examination skills [41]. They also found no difference in performance on a written test of related knowledge [41]. Dinh et al. evaluated the effect of adding point-of-care ultrasound to PE sessions in comparison to students in the prior year taught without ultrasound [25]. They found that ultrasound increased the number of students who received outstanding scores on an OSCE and that students and faculty had positive attitudes about including ultrasound in PE curricula.

The three other studies evaluated different technological approaches to preparing students for PE instructional sessions, including online modules and videos of how to perform PE exams [36,38,42]. Knutson et al. evaluated learner attitudes towards online modules as preparation for physical examination sessions [36]. They found that learners preferred an online module to reading a traditional textbook. Orientale et al. evaluated the effect of online videos as preparation material for PE sessions to a previous year without these sessions [42]. They found that this intervention improved learner performance of a head-to-toe checklist examination. Kurihara et al. conducted a four-arm randomized controlled trial comparing text book preparation, a computer-based module, and textbook reading plus a computer-based module to a control group as methods of preparing for PE sessions [38]. They found that all of the preparation methods improved learners’ performance on OSCE and multiple-choice tests, but found no difference between these interventions.

### Use of clinical experience in novice PE curricula

Four studies evaluated the effect of adding clinical encounters to PE curricula for novices [27,37,39,40]. Gradey et al. report a program where students evaluated nursing home patients as part of a PE curriculum [27]. They stated that the program was successful but provided no data in support of this claim. Marshal et al. described having students evaluate patients in ambulatory care settings as part of learning PE skills but provided no data on efficacy [39]. As part of an introductory PE course, McGlynn et al. compared the effect of having students evaluate patients in different settings. They had one group of students practice PE skills on hospitalized patients and compared their performance to students that practiced PE skills in outpatient clinics to see if this affected learning of PE skills [40]. They found no difference between the groups as evaluated by the performance of physical examination during a standardized patient encounter. Kossof et al. evaluated the effect of adding clinical encounters to pre-clinical PE instruction and found that it improved pediatric National Board of Medical Examiners shelf examination scores [37]. This change, however, paralleled a rise in MCAT scores between the control and intervention group. They found no difference in clinical performance or history and physical exam scores between the groups.

## Discussion

To our knowledge, this is the first systematic review of the literature on teaching PE to novice learners prior to 2015. The evidence we found for how to best teach PE skills to pre-clinical medical students is limited. With regards to our first question, ‘How are PE skills taught to preclinical medical students for the first time and what evidence supports these curricula?,’ we found that head-to-toe and organ system-based curricula were the only described methods. However, nearly half of the included studies did not describe the curriculum approach using this terminology, but rather a specific intervention added to an existing curriculum that was not described in detail. Additionally, none of the included studies described the theoretical framework supporting one approach versus the other.

With regard to our third question, ‘How do the head-to-toe and organ system-based curricula perform in comparison to each other?’ we were unable to find studies directly comparing these approaches. Previous systematic reviews of the PE literature have focused exclusively on PE as it relates to single organ systems [49–51]. The narrow focus of these approaches, and the emphasis placed on organ system exams within the PE literature in general, suggests the existence of an important but neglected area of inquiry. A study of 4^th^ year medical students by Wilkerson et al. demonstrated that many students are able to perform technically sound organ system-based exams, but lack the clinical insight to know when specific maneuvers are appropriate [52]. The current approach seems analogous to teaching a student driver how to operate a manual transmission without teaching when to shift gears (this is likely to lead to a bumpy ride). The recently described ‘core-and-cluster’ format may provide a way of integrating the head-to-toe and organ system-based approaches to teaching PE [53]. It also provides a scaffold for novice learners to decide which organ system-based exams to perform during a patient encounter. We found no articles during the time period we searched that evaluated this approach.

In answer to our fourth question, ‘What interventions, such as readings or online modules, have been applied to introductory PE curricula, and what evidence supports their use?,’ we found evidence to support the use of online or computer-based modules in addition to textbooks. Bedside ultrasound also shows promise as an adjunct to PE teaching. Finally, we found evidence supporting the use of non-faculty instructors for teaching PE skills to novices. These included residents, senior medical students, and PETAs. Use of non-faculty instructors offers a method of overcoming the barriers of cost and time constraints related to clinical faculty.

In the manuscripts we reviewed, we found two studies addressing the question of when in medical school PE skills should be taught to students [24,44]. Both studies found that first-year students showed superior performance on PE skills assessments in comparison to second-year students. The authors of one study ascribe this finding to the fact that the second-year students were taught using a curriculum with different content, and to the fact that these students were taught without PETAs [24]. The other article does not offer an explanation for this seemingly counterintuitive finding [44]. Choices surrounding clinical skills assessments, such as the setting and derivation sample for instrument development, would likely have a large impact on these findings. Without detailed descriptions of the assessments used in these studies, it is impossible to evaluate their validity.

### Limitations

Our search of the literature revealed only 32 studies that met our inclusion criteria, despite the ubiquity of PE teaching in medical schools. As such, our systematic review provides a limited view of the true state of affairs in PE teaching. The number of papers describing curricular innovations rather than outcomes also suggests the possibility of publication bias or else an underemphasis on curricular effectiveness.

We found a large reliance on learner attitudes and short-term skill assessments which provide only low-level evidence of learning. The objective measurements used most commonly were OSCEs, an evaluation strategy that can be problematic because it does not require integration of the individual components of clinical assessment [52]. The existing literature limits our ability to understand the effect that novice PE curricula have on future clinical performance. With the exception of two studies, the effects on students’ long-term learning and on clinical performance were not assessed [32,37].

The inconsistent description of assessment methodology and reliance on internally developed and non-validated assessments further limits the conclusions that can be drawn about curriculum effectiveness. The USMLE Step 2 Clinical Skills and multi-institution Clinical Performance Exams (CPX) exist as potential outcomes for clinical skills performance and include assessments of PE skills [7,54–56]. None of the included studies used one of these measures as an outcome.

### Implications

It is our opinion that consensus guidelines are needed for reporting the results from studies of PE curricula. Further descriptive work is also needed so that we can better understand how these skills are being taught, and what outcomes are being used at the national and international level. Assessments for different levels of learners, available to all institutions, can and should be developed. A recent national survey published by Uchida et al. sheds some light in this area [57]. In their survey of US medical schools, they found that 79% of the respondent schools teach physical examination early in the first year of medical school. The data they provide is an important first step in improving PE skills but much is left to be done.

Finally, those who teach PE skills to novices should be empowered to explore new techniques for teaching introductory clinical skills and for organizing the content in their courses. There is insufficient evidence supporting any existing method over another at this time. The core and cluster technique, recently described but developed for third-year medical students, presents a possible alternative [53,58]. It has the potential to bridge many of the gaps between the head-to-toe and organ system-based approaches and may provide for a smoother transition into clinical practice.

## Appendix A. Search terms and number of citations found for each database searched

**Table:**

Database	Search Terms	No. Articles
ERIC	‘physical examination’ AND ‘medical education’	612
SCOPUS	‘physical examination’ AND (medical student or undergraduate medical education)	1,090
And (teaching or learning)		
Medline	‘physical examination’ and (medical student or undergraduate medical education)	1,089
EMBASE	‘physical examination’ AND ‘medical education’ AND (teaching‘/exp OR curriculum OR Curriculum development) AND [english]/lim	571
PubMed		
	1) ‘physical examination’ [MeSH] AND (medical student or undergraduate) AND (teaching or learning)	1563
	2) ‘physical examination’ [MeSH] AND ‘education, medical’ [MeSH]	1961
	3) ‘physical examination’ AND “education, medical”[MeSH]	2400
	4) ‘physical examination’ AND (medical student or undergraduate) AND (teaching or learning) 2040	
Total citations		11,326
Duplicates		5908
**Individual articles for review**		**5418**

## Appendix B. Description of Curricula or intervention, Outcomes and Study Quality of 32 Included Studies in a Systematic Review of Physical Examination Curricula Used to Teach Novice Learners Published Through January 2015

**Table:**

First Author	Year	Country	MERSQI	Level of learners	Number of learners	Study Design	Intervention or Curriculum Summary	Outcome	Conclusion
Aamodt	2006	USA	7	MS1	175	Two group, non-randomized, non-concurrent	Use of physical exam teaching associates to teach physical examination curriculum	Learner Attitudes, cost analysis	Learners found the curriculum enjoyable and effective. The intervention was cost effective when compared to using faculty to teach physical examination skills.
Ainsworth	1991	USA	10	MS1	3000	Not described	15 years of experience using standardized patients with faculty facilitated small groups to teach physical examination skills	OSCE, no data reported	Description of curriculum only, no outcomes or research question.
Ali	2011	Pakistan	11.5	MS1	150	Two group, non-randomized, non-concurrent	Description of the introduction of a clinical skills course into pre-clinical training. Clinical skills curriculum used standardized patients with post graduate trainees as facilitators. No description of the specific exams taught.	OSCEs to evaluate clinical skills. Multiple choice questions to evaluate related knowledge.	Implementation of the curriculum improved medical knowledge and was associated with improved knowledge retention.
Alvarado	1998	Puerto Rico	8	MS2	45	Single group, non-randomized	Development of an organ-system based curriculum using standardized patients and peer practice with faculty facilitators.	Only musculoskeletal (MSK) data was collected. OSCE of MSK skills, learner attitudes	Only survey data was reported. Students rated the curriculum as effective.
Anderson	1978	USA	10	MS2	Experimental: 46, control: 41	Two group, non-randomized, concurrent	Use of physical exam teaching associates with abnormal physical exam findings teaching students physical examination skills compared to physician instructors teaching examination skills. Both groups taught in an organ system based curriculum.	OSCE, learner attitudes	Physical exam teaching associates resulted in superior learner satisfaction and superior performance.
Barley	2006	USA	11	MS1	Experimental: 71, control: 71	Two group, randomized controlled trial	Use of physical examination teaching associates to teach organ system based physical examination skills compared to physician taught physical examination skills.	OSCE	Students taught by physical exam teaching associates showed superior abdominal exam scores. For all other organ systems there was no significant difference.
Barnes	1978	USA	14	MS2	Experimental: 13, control: 14	Two group, randomized controlled trial	Comparison of fourth-year medical students as standardized patients teaching physical examination and history skills to students taught by faculty, resident or fellows teaching the skills.	OSCE, learner attitudes	No difference in OSCE outcomes. Initially student teachers resulted in higher learner satisfaction, at a follow up survey this difference lost significance.
Davidson	2001	USA	9	MS1 and MS2	Experimental: MS1 83, control MS2 118	Two group, non-randomized, concurrent. MS2 control group, MS1 intervention group.	Comparison of a second-year curriculum using faculty to teach a body area based approach to a first-year program using physical exam teaching associates to teach first year students a body area based approach.	OSCE, cost analysis	MS-1s taught by physical exam teaching associates showed improved scores on OSCEs in multiple body areas compared to MS-2s taught by faculty, physical exam teaching associates were less expensive by cost analysis.
Dinh	2015	USA	14	MS1	Experimental: 163, control: 138	Two group, non-randomized, non-concurrent	Comparison of faculty lead physical diagnosis labs with and without Ultrasound to teach an organ system based physical diagnosis course	OSCE, learner attitudes, instructor attitudes.	Ultrasound resulted in a significant improvement of OSCE scores. Learners reported ultrasound improved their ability to learn physical examination.
Frazer	1977	USA	4.5	MS1	Not reported	Not described	Use of trained standardized patients to teach an organ system based physical examination curriculum	Not reported	Authors report “sessions have been successful and rewarding.” Data not reported.
Grady	1990	USA	4.5	MS2	200	Not described	Addition of a program where students evaluate patients in a nursing home to a patient evaluation curriculum (curriculum not described)	Learner attitudes, oral quizzes and discussions with preceptors	Authors report “satisfaction with the program was high“, and ”oral quizzes and patient case discussions with the students convinced the instructors that this was certainly as effective a method as the traditional hospital-based approach.” Data not reported.
Haist	1996	USA	10	MS1	Not reported	Randomized controlled trial	Use of MS4 students to teach a head to toe physical examination checklist compared to students taught by faculty preceptors.	Learner attitudes, student facilitator attitudes	Data not reported. Authors report “using fourth-year students to teach first-year students was a success.”
Hamann	2002	USA	12.5	MS2	489	Single group, non-randomized, post test	Descriptive study of an organ system based clinical skills curriculum.	OSCE	Mean physical examination composite score of 65%.
Hardman	1997	Australia	7.5	MS3	60	Single group, non-randomized, crossover	Use of large group with artists models to teach physical examination skills. Curriculum not described further.	Learner attitudes, clinical assessment by Likert scale (not described)	Student response described as “satisfactory” (data not reported). Students taught by large group statistically outperformed those taught at the bedside on the clinical assessment.
Hasle	1994	USA	13	MS2	Experimental: 150, control: 150	Two group, non-randomized, non-concurrent	Use of physical exam teaching associated to teach an organ system based physical examination curriculum.	OSCE, learner survey, cost analysis	No difference in OSCE outcomes, Learners felt prepared, implementation resulted in a cost savings.
Jackson	2009	USA	11	MS2	Experimental: 2,606, control: 2,364	Two group, non-randomized, non-concurrent	Comparison of a structured organ system based clinical skills curriculum to a non-described previous curriculum.	MS 3 Clerkship evaluations	Implementation was associated with a statistically significant improvement in 9 of 12 domains evaluated in the third year.
Kim	2010	USA	8.5	MS1	108	single group, non-Randomized	Implementation of a voluntary practice session where MS2s reviewed a head to toe examination with MS 1 learners.	Learner and Instructor attitudes	Learners reported an improvement in comfort with skills. MS-2 students felt comfortable teaching near peers.
Kira	1998	Brazil	4.5	MS3	Not reported	Single group, non-randomized	Description of a introduction to clinical medicine course using an organ system based physical examination curriculum	OSCE, Learner attitudes	Students rated the course as effective. No learner evaluation data reported. No comparison group.
Klachko	1975	USA	10.5	MS1	107	Single group, non-randomized	Effect of teaching students a 55 maneuver head-to-toe examination which they had to memorize the checklist and list all the items in less than 10 minutes prior to examining patients.	Learner reported time to complete the patient examination during the MS-2 year. Learner survey	The curriculum decreased student reported time to perform a patient examination. Students reported the curriculum was useful.
Knutson	2005	USA	8	MS2	Not reported	3 group, randomized, crossover	Effect of adding having students prepare for organ system based physical examination small group sessions using a computer based tutorial on physical examination	Learner attitudes	Computer based tutorial received statistically higher ratings.
Kossoff	1999	USA	12	MS1 and MS2	Experimental: 200, control: 200	Two group, non-randomized, non-concurrent	Addition of an 18-month preceptorship to a pre-clinical clinical skills course	Clinical skills assessment scores, history and physical examination write-ups, NBME pediatrics Shelf examination scores, clerkship evaluations	Students taught with early clinical involvement showed superior Pediatrics Shelf examination performance. No other differences in outcomes found.
Kurihara	2004	Japan	12.5	MS3	Experimental: 3 arms of 15, control: 15	Four group, randomized controlled trial	RCT of 4 different methods of preparing students for physical examination sessions (content of sessions not described). Methods tested included textbook use, textbook and a computer based tutorial, computer based tutorial only and no preparation	OSCE, multiple choice question test, learner survey	All experimental arms performed better than the control group. No difference between any of the experimental arms. Subgroup analysis of low performers showed that computer based preparation was more effective for this group.
Marshal	1983	Canada	0.5	MS2	Not reported	Not described	Addition of ambulatory clinical experience to a pre-clinical, clinical skills course	OSCE	Not reported
McGlynn	1977	USA	12	MS2	Not reported	Two group, randomized controlled trial	Comparison of outpatient ambulatory care clinical experience vs inpatient experience as part of a clinical skills curriculum (physical exam sessions not described)	Performance of physical exam during a standardized patient encounter	No statistical difference between groups.
Mir	1984	UK	11.5	MS1	Experimental: 12, control: 13	Two group, randomized controlled trial	Comparison of using video recordings to small group instruction to teach introductory physical examination skills.	OSCE, multiple choice question test	No statistical difference between groups.
Orientale	2008	USA	12	MS1	Experimental: 158, control: 161	Two group, non-randomized, non-concurrent	Addition of videos showing the performance of maneuvers to a head-to-toe physical examination curriculum.	Head-to-toe checklist performance and Likert scale physical exam process assessment	Statistical improvement in performance of the head-to-toe checklist by learners who received the videos. No change in process scores.
Piryani	2013	Nepal	13	MS2	Experimental: 98, control: not reported	Two group, non-randomized, non-concurrent	Introduction of an organ system based physical examination curriculum where each organ system is taught by a clinical department.	OSCE and learner attitudes	The Curriculum was well liked. Implementation of the curriculum resulted in a statistical improvement in physical exam performance.
Rogers	2001	USA	11	MS1 and MS2	Experimental: 707, control: 596	Two group, non-randomized, non-concurrent	Movement of clinical skills curriculum from second year to first year.	OSCE	MS-1 students showed statistically better performance.
Stillman	1979	USA	13	MS1	Experimental: 46, control: 46	Three group, randomized controlled trial	Comparison of using faculty vs resident-physicians vs nurse practitioners to teach a head-to-toe physical examination course	Performance of a head-to-toe exam	No statistical difference between groups
Stillman	1981	USA	11.5	MS1	Not reported	Two group, non-randomized, concurrent	Comparison of two head-to-toe curriculums. One with more detailed checklists and more structure to the second with less detail and less structure	Performance of a head-to-toe exam using the more detailed checklist	Students taught using the more detailed checklist showed statistically superior performance.
Stillman	1997	China	15	MS3*	Experimental: 689, control: 178	Two group, non-randomized, non-concurrent	Development of a competency based clinical skills curriculum which included more organization, structured checklists and SP sessions compared to multiple baseline curricula. Specific changes included increased course hours, more hands-on practice, faculty development, “general exam” followed by organ system exams, improved assessments.	OSCE	Students taught using the new curriculum showed superior performance.
Wasson	1976	USA	10		Experimental: 6, control: 5	Two group, non-randomized, concurrent	Development of a curriculum where MS2 students teach MS1 students physical examination skills compared to MS3 students previously taught by faculty.	Performance of an history and physical on a standardized ambulatory patient. Students were rated on thoroughness.	MS-1 students were statistically superior.

***OSCE: Objective Structured Clinical Examination; MS-1: First-year medical student; MS-2 Second-year medical student; MS-3: Third-year medical student; MS-4: Fourth-year medical student**
